# Assessing infection control training in ICUs using the Kirkpatrick model: a prospective cohort study

**DOI:** 10.1186/s13756-025-01587-6

**Published:** 2025-06-09

**Authors:** Sahar Elsheikh, Afaf Magdy, Lamiaa Asem

**Affiliations:** 1 Nursing Department, Shebin Elkom Fever Hospital, Ministry of Health, Menoufia, Egypt; 2 Clinical Pharmacy Department, Shebin Elkom Fever Hospital, Ministry of Health, Menoufia, Egypt

**Keywords:** Infection prevention, Kirkpatrick model, Hospital-acquired infections, Healthcare training, Compliance

## Abstract

**Background:**

Hospital-acquired infections (HAIs) are a significant global health challenge, particularly in intensive care units (ICUs), where patient vulnerability is high. Effective infection prevention and control (IPC) training is critical for reducing HAIs and improving healthcare outcomes. This aims to evaluate the efficacy of an IPC training program via Kirkpatrick’s four-level model.

**Methods:**

A prospective cohort study was conducted between June and December 2024 at Shebin El Kom Fever Hospital’s ICU. The study involved 106 healthcare workers (84 nurses, 22 physicians) who participated in a two-month training program combining theoretical lectures and practical sessions. Program effectiveness was assessed via Kirkpatrick’s 4 levels: reaction (satisfaction surveys), learning (knowledge tests), behavior (direct observation), and results (clinical outcomes).

**Results:**

Ninety health care workers (HCWs) whose level of satisfaction exceeded 80% across all training aspects (Level 1) were included. The knowledge assessment revealed a significant improvement in the mean test score from 76.93 to 82.29% (*p* = 0.0112) (Level 2). Behavioral evaluation revealed substantial improvements in infection control practices, particularly in nurses’ aseptic procedures (40.00–83.54%, *p* < 0.001) and physicians’ personal protective equipment (PPE) usage (19.05–62.50%, *p* = 0.0391) (Level 3). At Level 4, no significant changes were observed in HAIs, mortality rates, or hospital stay costs.

**Conclusion:**

IPC training programs significantly enhance HCW knowledge and compliance with infection control practices, laying the groundwork for sustainable ICU infection control. While immediate improvements in hospital metrics were not observed, long-term monitoring is crucial to achieving full benefits. Enhanced compliance may reduce HAIs and associated costs over time.

**Supplementary Information:**

The online version contains supplementary material available at 10.1186/s13756-025-01587-6.

## Background

Hospital-acquired infections (HAIs) are critical global public health concerns that significantly affect both patient outcomes and healthcare economics [1, 2]. These infections are particularly prevalent in intensive care units (ICUs), where approximately 30% of patients experience at least one HAI episode during their hospital stay [3, 4]. This high susceptibility stems from the frequent use of invasive procedures, the severity of patients’ conditions, and the presence of immunocompromised or vulnerable populations such as elderly individuals and neonates [4].

HAIs also contribute to increased hospital stays, medication costs, equipment use, and the global burden of antimicrobial resistance [3, 5–7].

Preventing HAIs requires comprehensive infection prevention and control (IPC) strategies. These evidence-based practices are designed to minimize the transmission of infectious agents within healthcare facilities and involve coordinated efforts across all levels—from hospital leadership to frontline staff [6].

International organizations such as the World Health Organization (WHO) and the Centers for Disease Control and Prevention (CDC) have developed detailed frameworks for IPC implementation, emphasizing key components such as hand hygiene, the use of personal protective equipment (PPE), aseptic techniques, and environmental sanitation [8, 9].

A critical element in the effectiveness of IPC programs is the training of healthcare workers (HCWs), who play a central role in ensuring adherence to infection control measures [10–13].

Well-structured training enhances compliance and promotes a safety-oriented culture. However, training implementation varies significantly across healthcare systems, especially in low- and middle-income countries (LMICs) [11].

A systematic review highlighted that compliance with IPC practices is influenced by the clarity and practicality of guidelines, communication strategies, and training availability [14].

Evidence from regional studies further supports this point. A study conducted in a large academic hospital in Egypt revealed that a structured IPC training course significantly improved the knowledge and practices of medical interns, illustrating the positive impact of organized training programs [15]. Additionally, a multicountry qualitative study involving stakeholders from Saudi Arabia, Pakistan, India, Indonesia, the Philippines, and Australia revealed that while IPC training was mandatory in many settings, its implementation was inconsistent. Classroom-based instruction was the dominant format, with limited use of online platforms. Despite improvements in training during the COVID-19 pandemic, many regions still face challenges in terms of delivery capacity [16].

However, training alone is insufficient unless its outcomes are systematically evaluated. The Kirkpatrick model is the most widely adopted framework for assessing training effectiveness. This comprehensive model comprises four sequential levels: Level 1 (reaction) assesses participants’ satisfaction with the training; Level 2 (learning) measures knowledge acquisition; Level 3 (behavior) evaluates changes in workplace practices; and Level 4 (results) examines the overall organizational impact [17–19].

Despite the widespread use of IPC training, few studies have comprehensively assessed its effectiveness across all four Kirkpatrick levels, particularly in ICU settings in LMICs. This represents a significant research gap, as understanding these outcomes is essential to ensure the success of training programs and their contribution to HAI reduction [20].

## Research question and objectives

### Primary objective

This study aimed to evaluate the impact of a structured infection prevention and control (IPC) training program on the knowledge, behavior, and clinical outcomes of healthcare workers (HCWs) in the intensive care unit (ICU).

### Research question

Does a structured IPC training program improve knowledge and adherence to IPC practices and reduce healthcare-associated infections (HAIs) among ICU healthcare workers?

## Materials and methods

### Study design and setting

A prospective cohort study was conducted between June 2024 and December 2024 in the intensive care unit (ICU) of Shebin El-kom Fever Hospital. The ICU consists of both adult ICU and pediatric ICU (PICU) sections, with a total of 20 beds. The unit maintained a 1:1 nurse-to-patient ratio and a 1:4 sink-to-patient-bed ratio.

At our hospital, the annual training plan includes a periodic infection prevention and control (IPC) training program conducted every six months by the IPC hospital team to cover all healthcare workers (HCWs). This study aimed to address the increasing rates of hospital-acquired infections (HAIs) in the ICU observed before the study period and assess the knowledge and behavior of HCWs, which may have contributed to this increase.

### Study participants

The sample size calculation was based on an interventional prospective study using Stata 17 [20]. The primary outcome measure was knowledge improvement as assessed by posttraining test scores. A 14% improvement in knowledge level was targeted as the expected effect size. With a statistical power of 80% and a two-sided significance level of 0.05, a minimum sample of 60 participants (30 per group) was deemed adequate for detecting this difference. To account for potential dropouts, an additional 15% was added, resulting in a target enrollment of 70 participants.

The study recruited all HCWs involved in direct patient care in the ICU, totaling 106 participants: 84 nurses (both registered and enrolled) and 22 physicians (intensive care specialists and pediatricians), representing the full ICU medical staff. HCWs without direct patient contact, those who did not complete the training program, and housekeeping staff were excluded from the study.

#### The study was conducted in three distinct phases

**Fig. 1:**
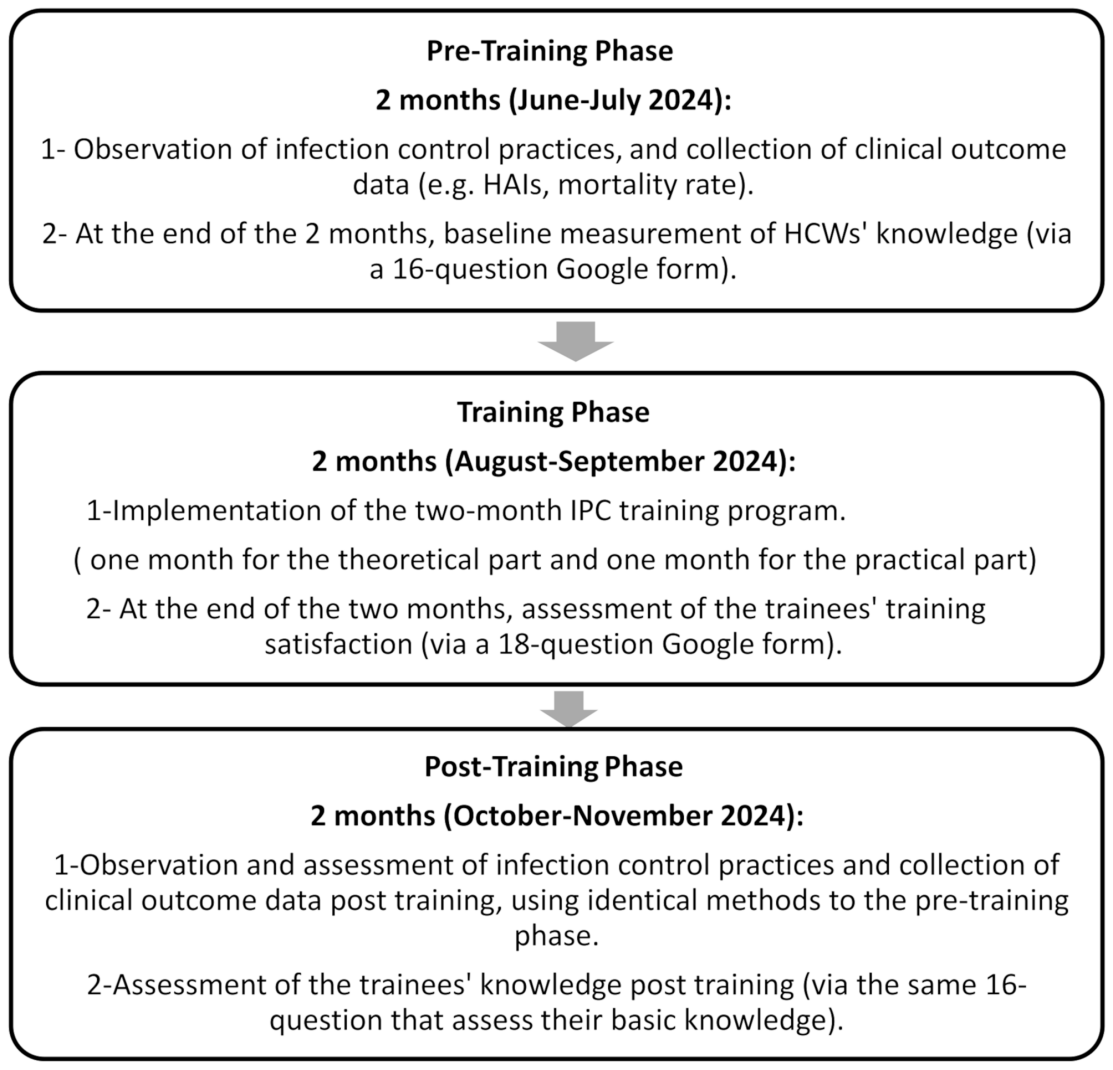
Study timeline schematic diagram

the first two months involved recording HCWs’ adherence to IPC measures before training and assessing their knowledge by sending 16 questions on a Google form, followed by a two-month training program. In the final two months, knowledge and adherence among the same HCWs were recorded posttraining (Figure 1 below).

### Training program

The study involved a two-month IPC training program, which consisted of both theoretical and practical components. A summary of the training program is provided in Table 1 below.

**Table 1:** Summary of the infection prevention and control training Program:

Component	Description
Duration	Two months
Participants	106 healthcare workers (84 nurses, 22 physicians)
Theoretical Component	- 3 presentations on Egyptian IPC Guidelines - 5 lectures covering infectious processes, nosocomial infections, transmission modes, and WHO standard precautions - Distribution of IPC guideline handouts
Practical Component	- Daily 2-hour ICU practical sessions - Hands-on training on IPC practices: hand hygiene, PPE use, environmental cleaning, instrument sterilization, sharp handling, waste disposal, injection practices, patient isolation
Educational Materials	- IPC guideline handouts - Posters on hand hygiene techniques, 5 moments of hand hygiene, and PPE donning/doffing procedures
Practical Training Format	- Trainees divided into 15 groups - Conducted by the hospital IPC team - Trainer demonstration followed by trainee application

The theoretical component “oral presentation including 3 presentations discussing the guidelines from the Egyptian Infection Prevention and Control Guidelines [21]” included five lectures covering infectious processes, nosocomial infection definitions, transmission methods, and WHO standard precautions [7, 22]. The practical component involved daily two-hour sessions in the ICUs (training sessions on top of work) focused on IPC components, including hand hygiene (HH), wearing PPE, environmental surface cleaning, instrument sterilization, safe sharp handling, waste disposal, injection practices, and patient isolation. Additionally, Egyptian Infection Prevention and Control Guidelines [21] were given to all the participants as a handout, including instructions about IPC application measures. Posters about the HH technique, 5 moments of HH, and the technique of putting and doffing PPE are distributed in the ICU by the IPC team as part of educational material to the HCWs [23]. The IPC team organizes and delivers the training sessions, and the trainees are divided into 15 groups. During each session, the trainer performed IPC components in front of the trainee, and then each trainee applied the same component in front of the trainer.

### Evaluation methods

1. **Level 1 (**reaction): This level was assessed through a Google form questionnaire evaluating training material, instructor effectiveness, and the training environment. The questionnaire was developed according to the guidelines of the WHO [22]. The participants were asked to score 18 questions as “Strongly disagree”, “Disagree”, “Strongly agree”, or “Agree”. The last section comprised 2 open-ended questions probing for information. The first question was to rate the training class (low or high) from 1 to 5. Participants were also asked if they were interested in participating in the upcoming training (see Additional file 1).
2. **Level 2 (Learning)** was measured via the same multiple-choice 16-question that was asked before training (pre- and posttest) on a Google form, with each question carrying one mark with a total of 16 marks (see Additional file 2). The content validity of the questionnaire was confirmed by a team of IPC experts.

### Level 3– behavior (observation protocol)

To ensure consistency and reliability in data collection, the observer was an independent, qualified registered nurse who had been trained (had an IPC certificate) and had accumulated experience in patient care, which ensured that the observer was familiar with the ICP guidelines. The observer underwent a two-week calibration period during which their assessments were validated by the IPC team leader. During this period, independent assessments were conducted on the same HCWs to ensure consistency. Once a > 90% agreement rate was achieved on three consecutive joint observations, the observer was deemed reliable. To maintain consistency, the same observer conducted all observations throughout the study period.

HCWs’ adherence to IPC measures was evaluated through direct observation. Nurses were observed in four specific situations: central venous pressure (CVP) instillation, drug administration, care for ventilated patients, and general patient care. The observations focused on several IPC components, including hand hygiene, PPE usage, sharp equipment handling, decontamination of equipment, waste management, handling of linens, and environmental cleaning.

Observations were carried out during morning shifts every day of the week (except Fridays) for two months. To minimize the Hawthorne effect, the observer did not interrupt the care procedures.

Informed consent was obtained from the participants but without disclosure to the observing person.

The observation checklist, developed by infection control experts, was based on WHO, CDC, and Egyptian Infection Prevention and Control Guidelines [21, 22]. It incorporated the WHO Five Moments of HH checklist (observation form) [23] and recorded the duration of HH actions. Compliance was calculated as the ratio of performed actions according to the number of opportunities. Other items included putting on and doffing PPE, cleaning and disinfection of environmental surfaces, sterilization of all medical devices, safe handling and disposal of sharps, safe handling of laundries, safe disposal of waste, and isolation of infected patients. The items observed were scored as one (1) for applied procedures and zero (0) for nonapplied procedures.

### Level 4– Results (Outcomes Assessment)

Level 4 assessed outcome measures before and after the training program by comparing pre- and posttraining rates of hospital-acquired infections (HAIs) (defined as infections occurring more than 48 h after hospitalization) [22]. These included bloodstream infections (BSIs), central line-associated bloodstream infections (CLABSIs), catheter-associated urinary tract infections (CAUTIs), and ventilator-associated pneumonia (VAP).

Additionally, we evaluated patient care costs, clinical improvement, and mortality rates. Clinical improvement was assessed through various indicators: reduction or elimination of symptoms such as pain, fever, or inflammation (symptom relief); restore the patient’s ability to perform daily activities (functional recovery); and normalization of clinical parameters, including blood tests and imaging results (laboratory and diagnostic findings) [24]. The mortality rate was calculated as follows: number of deaths due to a disease divided by the total population [25].

### Statistical analysis

The statistical analysis included descriptive statistics for the study variables and demographic data. Paired t tests were used to compare pre- and posttraining learning outcomes. McNemar’s test was used to assess changes in behavior and compliance with infection control measures. Chi-square tests or Fisher’s exact tests were used to evaluate changes in HAI rates and to compare the mortality rate and clinical improvement rate before and after training. T tests were used to compare patient costs. All analyses were performed via Stata 17.

## Results

A total of 106 HCWs, including 84 nurses and 22 physicians, participated in the study. The mean age was 29.06 ± 2.78 years for nurses and 36.59 ± 6.31 years for physicians. Most nurses (86.90%) had 1–10 years of experience. Most nurses were female (80.95%), whereas physicians had a more balanced gender distribution (54.55% male).

### Kirkpatrick level 1: reaction (Participant Satisfaction)

For the Kirkpatrick Level 1 evaluation, 90 participants completed the training feedback questionnaire. The participants’ level of satisfaction with the instructor’s knowledge and preparedness was 93.33%, and the instructor’s presentation received 95.55% satisfaction. A total of 95.55% of the participants reported that the training material met their needs, and 97.78% were satisfied that the knowledge they gained from the training applied to their work. The participants’ satisfaction with the training environment and facilities was 93.33%.

A total of 88.89% of the participants expressed interest in participating in future training sessions (Table 2).

**Table 2:** Participant satisfaction with infection control training (Level 1: Reaction)

Aspect Evaluated	Satisfied/Very Satisfied (%)
The instructor was knowledgeable and well-prepared for all topics presented	93.33
The instructor knows the material and presented the material in an organized manner	95.55
Training met needs	95.55
Knowledge applicable to work	97.78
Training environment satisfaction	93.33
Would participate in future training	88.89
Overall rating of class (mean of rating grade)	76.67%

### Kirkpatrick level 2: learning (knowledge improvement)

In the Level 2 evaluation, a comparison of the pre- and posttraining test scores revealed a significant increase in the mean final grade from 12.31 ± 1.74 to 13.17 ± 1.21 (*p* = 0.0112), representing an improvement from 76.93 to 82.29% (Table 3).

**Table 3:** Pre- and posttraining knowledge scores (Level 2: Learning)

Assessment	Pretest (*n* = 42)	Posttest (*n* = 56)	Test	*P* value
Final Grade (Mean ± SD)	12.31 ± 1.74	13.17 ± 1.21	2.657	0.0112
Mean of Grade Percentage % (Mean ± SD)	76.93 ± 10.91	82.29 ± 7.90	2.657	0.0112

### Kirkpatrick level 3: behavior (compliance with infection control measures)

The Level 3 (behavior) evaluation revealed significant improvements in nurses’ key infection control practices. Compliance with HH before performing aseptic procedures increased significantly from 40.00% before training to 83.54% after training (*p* < 0.001). Similarly, compliance after contact with body fluids significantly improved from 37.88 to 84.81% (*p* < 0.001), as did compliance after contact with inanimate objects, which increased from 39.71 to 82.28% (*p* < 0.001), but compliance before touching patients decreased from 7.14 to 3.80%, but the difference was not significant (*p* = 0.508).

For environmental cleaning, compliance with cleaning surfaces once per shift improved significantly from 46.48 to 72.37% (*p* = 0.005). In sterilization practices, the number of correct methods for surgical equipment sterilization increased from 73.53 to 89.87% (*p* = 0.012), and compliance with safe injection practices improved from 38.64 to 64.06% (*p* = 0.0034). Additionally, compliance with glove removal increased significantly from 75.32 to 97.06% (*p* = 0.0129).

Likewise, wearing PPE correctly (22.62% vs. 36.49%, *p* = 0.087) and safe handling of waste (94.05% vs. 96.20%, *p* = 0.2891) showed no statistically significant improvements despite numeric differences (Table 4a).

**Table 4a:** Behavioral compliance with infection control practices among nurses (Level 3: Behavior)

Infection Control Measures	Compliance Before Training (%)	Compliance After Training (%)	McNemar’s Test	*P* value
Hand hygiene before aseptic procedure	40.0%	83.54%	26.13	< 0.001
Hand hygiene after contact with body fluids	37.88%	84.81%	26.95	< 0.001
Hand hygiene before touching patient	7.14%	3.80%	1.00	0.508
After touching patient	43.24%	41.77%	0.00	1.000
after contact with inanimate objects	39.71%)	82.28%	25.00	< 0.001
Clean surface with visible secretion	48.00%	72.37%	4.45	0.065
cleaning surface once in shift	46.48%	72.37%	8.53	0.005
sterilization of surgical equipment’s:	73.53%	89.87%	7.20	0.012
Safe injection practices	38.64%	64.06%	9.31	0.0034
safe handling of sharps	50.88%	56.25%	2.58	0.1671
Removal of gloves between patients	75.32%	97.06%	7.14	0.0129
Wearing PPE correctly	22.62%	36.49%	3.57	0.087
Safe handling of waste	94.05%	96.20%	2.00	0.2891

PPE: Personal protective equipment

Among the physicians, significant improvements were observed in two key areas: HH after touching patients increased from 55.00 to 86.36% (*p* = 0.016), and correct use of PPE improved from 19.05 to 62.50% (*p* = 0.039). (Table 4b).

**Table 4b:** Behavioral compliance with infection control practices among physicians (Level 3: Behavior)

Infection Control Measures	Compliance Before Training (%)	Compliance After Training (%)	McNemar’s test	*P* value
Hand hygiene before touching the patient	4.76%	4.55%	0.00	1.000
Hand hygiene before performing an aseptic procedure	66.67%	95.45%	2.00	0.5
Hand hygiene after contact with body fluids	71.43%	95.45%	2.00	0.500
Hand hygiene after touching the patient	55.00%	86.36%	7.00	0.016
Hand hygiene before touching inanimate objects	71.43%	95.45%	2.00	0.500
Wearing PPE correctly	19.05%	62.50%	5.44	0.039
Safe handling of sharps	40.00%	72.73%	1.00	1.000
Safe injection practices	60.00%	80.00%	1.00	1.000
Safe handling of waste	95.24%	100.00%	1.00	1.000
Removal of gloves between patients	89.47%	81.82%	0.33	1.000
Aseptic device management	33.33%	72.73%	1.00	1.000
Patients isolation	4.76%	31.82%	--	1

PPE: Personal protective equipment

### Kirkpatrick level 4: results (clinical outcomes)

For Level 4, the study compared outcomes between the pretraining (131 patients) and posttraining (147 patients) periods. None of the clinical outcomes showed statistically significant changes. The HAI rates remained statistically similar between the pretraining (1.53%) and posttraining (2.72%) periods (*p* = 0.687). Similarly, no significant differences were observed in mortality rates (25.95% vs. 21.09%, *p* = 0.395), and these mortality rates were not adjusted for the severity of cases. Clinical improvement rates (60.31% vs. 61.90%, *p* = 0.806) and septic shock incidence rates (16.03% vs. 16.33%, *p* = 1.000) were reported. The mean cost of care also remained statistically similar before (14,138.79 ± 74,796.82 LE) and after training (13,253.45 ± 14,534.19 LE, *p* = 0.879) (Table 5).

**Table 5:** Clinical outcomes before and after training (Level 4: Results)

Result	Before Training (%)	After Training (%)	Test Value	*P* value
HAIs Overall	1.53%	2.72%	χ²=0.4679	0.687
CRE Infections	1.53%	2.04%	χ²=0.1037	1.000
CRAB Infections	0.00%	0.68%	χ²=0.8944	1.000
Mortality Rate	25.95%	21.09%	χ²=0.9155	0.395
Clinical Improvement	60.31%	61.90%	χ²=0.0746	0.806
Septic Shock	16.03%	16.33%	χ²=0.0045	1.000
Mean Care Cost (LE)*	14,138.79 ± 74,796.82	13,253.45 ± 14,534.19	t = 0.151	0.879

χ²=Chi-square test, t = t test

*Note: Mean Care Cost values are presented as the mean ± standard deviation in the Egyptian Pounds (LE). HAIs = healthcare-associated infections; CRE = carbapenem-resistant Enterobacteriaceae; CRAB = carbapenem-resistant *Acinetobacter baumannii*

## Discussion

Hospital-acquired infections remain a significant challenge in ICUs, leading to increased morbidity, mortality, and healthcare costs. Training HCWs in IPC is crucial for reducing HAI rates. Our study comprehensively evaluated an IPC training program via the Kirkpatrick four-level model, assessing not only the immediate impact on HCWs’ knowledge and satisfaction but also behavioral changes and organizational outcomes.

The key findings revealed positive trainee reactions (Level 1), improved knowledge scores (Level 2), and enhanced compliance with several IPC measures (Level 3), although with mixed results in some areas, whereas the Level 4 outcomes did not significantly change during our observation period.

At Level 1 (reaction), the training program received positive feedback, with more than 80% of the respondents expressing satisfaction. The instructor’s knowledge and presentation style were particularly well received, with 81.11% agreeing that material was presented in an organized manner. This high satisfaction rate is crucial, as positive learner reactions are considered a fundamental prerequisite for effective learning, as established by previous studies by Savul et al. (2021) [26] and Gebrehiwot & Elantheraiyan (2023) [27], who reported comparable satisfaction levels in their train-trainer programs. The positive reaction likely stems from our comprehensive curriculum design combining both theoretical and practical components and the use of hand-out demonstrations, which participants often find more engaging than do purely didactic approaches.

The Level 2 evaluation demonstrated a statistically significant improvement in knowledge scores, increasing from 76.93 to 82.29%. This improvement is particularly meaningful given the participants’ relatively high baseline knowledge. Similar improvements were reported by Faraz et al. (2024) [28], who reported substantial improvement in nurses’ knowledge following IPC training. This aligns with results from the targeted infection prevention (TIP) study by Koo et al. (2016) [29], which reported significantly improved knowledge scores among nursing home healthcare personnel.

The Level 3 (Behavior) evaluation revealed mixed results in translating knowledge into practice. Among nurses, significant improvements occurred in several critical areas, with HHs before aseptic procedures increasing from 40 to 83.54% and proper handling of body fluids improving from 37.88 to 84.81%. These substantial improvements in key infection prevention behaviors are particularly important given their direct impact on patient safety. The compliance rate of HHs before touching patients decreased from 7.14 to 3.80% (*p* value 0.508), and the compliance rate was low before starting training. This may be due to the improper attitudes of HCWs, as many HCWs prefer to wear gloves rather than hand washing and may lack knowledge that alcohol hand rubbing may be an alternative to hand washing with soap and water. After training, HH compliance before touching the patient decreased, which may be due to the clinical condition of the patients, who sometimes need multiple procedures and quick decisions, which is consistent with the findings of Mahfouz et al. (2013) [30]. A previous study reported that taking action before contact with a patient is considered an important risk factor for noncompliance with hand hygiene [31].

Among physicians, significant improvements were observed in wearing PPE correctly (19.05–62.50%) and HH after patient contact (55–86.36%), whereas other metrics showed nonsignificant improvements [31, 32]. Previous studies have demonstrated that HCWs often lack basic IPC knowledge and skills, with significant improvements observed after training. Factors influencing adherence to IPC guidelines include workplace culture, managerial support, physical space, access to protective equipment, and a desire to provide quality patient care [33]. To enhance IPC practices, a multifaceted approach, including regular, structured training programs, supervision, and creating an enabling environment in healthcare settings, is recommended [14].

Despite these behavioral improvements, the Level 4 evaluation revealed no significant changes in HAIs, mortality rates, or hospital stay costs. HAIs are influenced by multiple elements, including infrastructure limitations and resource constraints, which may hinder the effectiveness of IPC programs. Similar challenges were reported by Kakkar et al. (2021) [34], who reported that a single educational module improved nurses’ knowledge and attitudes but did not significantly reduce IV line infection (*P* = 0.15) or CAUTI rates. Another study demonstrated that increased HH compliance led to decreases in infections caused by *MRSA*, *Pseudomonas aeruginosa*, and *Acinetobacter baumannii* but failed to significantly affect ventilator-associated pneumonia, CAUTIs, or antibiotic consumption [35]. Conversely, a meta-analysis of 11 studies (2014–2024) revealed that IPC measures significantly reduce HAIs and improve compliance among HCWs [36].

With respect to multidrug-resistant organisms (MDROs), we observed minimal changes in infection rates. While not statistically significant, the maintenance of stable MDRO rates despite increasing infection admissions could be interpreted positively, particularly given the rising global challenge of antimicrobial resistance.

Importantly, our findings regarding organizational outcomes (level 4) may be limited by the relatively short posttraining observation period of two months. Research has suggested that sustainable changes in HAI rates often require longer monitoring periods. A study conducted at Al-Azhar New-Damietta University Hospital in Egypt assessed the impact of an intervention training program on HAI rates in ICUs over 15 months. The findings demonstrated significant reductions in HAI rates, highlighting that sustainable changes often require extended monitoring periods to become evident [20]. Therefore, our two-month posttraining observation period may have been insufficient to capture the full impact of the behavioral changes on clinical outcomes.

### Study limitations

Our study has several important limitations that should be considered when interpreting the results.

First, the short follow-up period of only two months posttraining was likely insufficient to detect meaningful changes in healthcare-associated infection (HAI) rates or other organizational metrics. Long-term improvements in infection control usually require ongoing support and reinforcement, but the program lacked sustainability strategies or follow-up plans. Without continued guidance, the behavioral improvements observed during the study may not have persisted beyond the intervention period.

Second, the daily presence of an observer in the ICU may have influenced healthcare workers’ (HCWs’) behavior—a potential Hawthorne effect—leading to changes in performance during observation.

Third, not all the procedures targeted for observation may have occurred during the observation windows, limiting the completeness of data collection.

Fourth, the small sample size of physicians limits the ability to generalize the findings to a broader population.

Finally, the single-center design limits the generalizability of our findings to other healthcare settings.

### Implications for practice

These findings show that ongoing IPC training and monitoring are important for keep improving behavior. Although there was no major improvement in Level 4 metrics during the study, better adherence to IPC measures can reduce HAIs and related costs over time. Future training should include reference courses, Continuous support and regular feedback help maintain changes in behavior. There should also be follow-up assessments and more training to build on early improvements. Strong support from the organization, with enough resources and Leadership commitment is essential for better patient outcomes. Future studies should look at ways to remove barriers to effective IPC and use larger, multi center studies to make the results more widely applicable. Researchers should also examine how system-level changes, such as better infrastructure and technology, can work with training. Finally, ways to sustain improvements, such as Ongoing education and reinforcement will be important for long-term success in IPC compliance.

## Conclusion

This study demonstrated that IPC training programs significantly improved HCW knowledge and compliance with essential infection control practices. While immediate improvements in hospital metrics were not observed, these behavioral changes represent critical progress toward achieving sustainable infection control in ICUs. Future interventions should address the identified limitations by incorporating longer follow-up periods and structured sustainability strategies. Additionally, multimodal approaches that combine education with system-level changes may be necessary to achieve significant improvements in clinical outcomes. Long-term monitoring and support are essential to realize the full benefits of such programs.

## Electronic supplementary material

Below is the link to the electronic supplementary material.

Additional file 1

Additional file 2

## Data Availability

No datasets were generated or analysed during the current study.

## Abbreviations

PPE: Personal protective equipment
HAIs: Hospital-acquired infections
HCWs: Healthcare workers
IPC: Infection prevention and control
ICUs: Intensive Care Units
CLABSI: Center Line-associated Blood Stream Infection
CAUTI: Catheter-associated urinary tract infection
VAP: Ventilator-associated pneumonia
MRSA: methicillin-resistant Staphylococcus aureus
CVP: Central venous pressure
WHO: World Health Organization
CDC: Centers for Disease Control and Prevention
LMICs: low- and middle-income countries
TIP: targeted infection prevention
MDROs: multidrug-resistant organisms
