# Supplementary material for: Assessing infection control training in ICUs using the Kirkpatrick model: a prospective cohort study
Source: Antimicrob Resist Infect Control. 2025 Jun 9;14:65. doi: 10.1186/s13756-025-01587-6 (PMC12150569; doi:10.1186/s13756-025-01587-6)
Supplement: Supplementary file 1 — Additional file 1 [file 13756_2025_1587_MOESM1_ESM.docx]

**Kirkpatrick Level 1: Reaction (Participant Satisfaction)**

**The training feedback questionnaire:**

| **Questions** | **Strongly disagree** | **disagree** | **Agree** | **Strongly agree** |
| --- | --- | --- | --- | --- |
| **For the instructor** | | | | |
| The instructor knows the material and presented the material in an organized manner | **2 (2.22%)** | **2 (2.22%)** | **73 (81.11%)** | **13 (14.44%)** |
| The instructor was responsive to the participants | **2 (2.22%)** | **3 (3.33%)** | **63 (70%)** | **22 (24.44%)** |
| The instructor was knowledgeable and well-prepared to all topics presented | **4 (4.44%)** | **2 (2.22%)** | **65 (72.22%)** | **19 (21.11%)** |
| The instructor's style and delivery was effective | **2 (2.22%)** | **3 (3.33%)** | **66 (73.33%)** | **19 (21.11%)** |
| The instructor provided adequate audio / visual aids | **2 (2.22%)** | **8 (8.89%)** | **69 (76.67%)** | **11 (12.22%)** |
| **2- For the training program** | | | | |
| The training met your needs | **2 (2.22%)** | **2 (2.22%)** | **74 (82.22%)** | **12 (13.33%)** |
| you found the subject matter relate to your work duties and requirements | **2 (2.22%)** | **5 (5.55%)** | **63 (70%)** | **20 (22.22%)** |
| The training was of adequate length for the topics presented | **2 (2.22%)** | **4 (4.44%)** | **74 (82.22%)** | **10 (11.11%)** |
| You will be able to use the knowledge gained from this training | **0 (0%)** | **2 (2.22%)** | **71 (78.89%)** | **17 (18.89%)** |
| The training objectives were identified and meet | **0 (0%)** | **2 (2.22%)** | **73 (81.11%)** | **15 (16.67%)** |
| The presentation was organized and contributed to your knowledge | **0 (0%)** | **3 (3.33%)** | **74 (82.22%)** | **13 (14.44%)** |
| Class participation was encouraged | **0 (0%)** | **4 (4.44%)** | **74 (82.22%)** | **12 (13.33%)** |
| **3- For the environment** | | | | |
| The facility provided a comfortable learning environment | **1 (1.11%)** | **5 (5.55%)** | **73 (81.11%)** | **11 (12.22%)** |
| Breaks were provided when needed and were of adequate length | **1 (1.11%)** | **11 (12.22%)** | **71 (78.89%)** | **7 (7.78%)** |
| Did the learner feel comfortable in the surroundings | **1 (1.11%)** | **3 (3.33%)** | **74 (82.22%)** | **12 (13.33%)** |
| Would you recommended this training to other employees | **1 (1.11%)** | **1 (1.11%)** | **75 (83.33%)** | **13 (14.44%)** |
| How do you rate this training class | **Mean = 76.67%** | | | |
| Would you like participate in upcoming trainings | **88.89%** | | | |
