# Supplementary material for: Assessing infection control training in ICUs using the Kirkpatrick model: a prospective cohort study
Source: Antimicrob Resist Infect Control. 2025 Jun 9;14:65. doi: 10.1186/s13756-025-01587-6 (PMC12150569; doi:10.1186/s13756-025-01587-6)
Supplement: Supplementary file 2 — Additional file 2 [file 13756_2025_1587_MOESM2_ESM.docx]

**Kirkpatrick Level 2: Learning (knowledge improvement)**

**Infection control questionnaire:**

| **Question** | **Pre-test**  **(n=42)** | **Post- test (n=42)** | **test** | **P value** |
| --- | --- | --- | --- | --- |
| Washing time for simple hand washing is 30 seconds | **12 (28.57%)** | **13 (30.95%)** | **0.07** | **1.000** |
| It is not necessary to wear sterile gloves when preparing and mixing IV fluids | **30 (71.43%)** | **35 (83.33%)** | **1.92** | **0.267** |
| 3- Heavy duty gloves are used to deal with blood spills | **25 (59.52%)** | **23**  **(54.76%)** | **0.14** | **0.851** |
| 4- When installing a urinary catheter, wash your hands with simple washing only | **37 (88.09%)** | **39 (92.86%)** | **0.67** | **0.687** |
| 5- Betadine at a concentration of 7.5% is used to disinfect the patient’s skin | **18**  **(42.86%)** | **27**  **(64.28%)** | **3.86** | **0.0784** |
| 6- The average duration for changing the cannula is 3-4 days | **36**  **(85.71%)** | **32**  **(76.19%)** | **1** | **0.454** |
| 7- The urinary catheter is changed 14 days after its installation | **15**  **(35.71%)** | **24**  **(57.14%)** | **3.24** | **0.108** |
| 8- Tongue depressors and intravenous devices are disposed of in the safety box | **39**  **(92.86%)** | **42**  **(100.0%)** | **3.00** | **0.250** |
| 9- When dealing with cases of TB, it is not necessary to wear N95 face mask | **34**  **(80.95%)** | **36**  **(85.71%)** | **0.33** | **0.774** |
| 10- When dealing with isolation cases, personal protective equipments are removed in the following order: gloves - face shield - gown - and the mask is removed after leaving the room, rubbing with alcohol every time the PPEs are removed. | **40**  **(95.24%)** | **40**  **(95.24%)** | **0.00** | **1.000** |
| 11- Unused supplies that are present in the patient’s area are not disposed of and are returned to the supplies cupboard. | **42**  **(100.0%)** | **42**  **(100.0%)** | **-** | **1.000** |
| 12- The expiration date of the multi-dose medication package is calculated after opening it according to the manufacturer’s instructions | **41**  **(97.62%)** | **41**  **(97.62%)** | **0.00** | **1.000** |
| 13- It is necessary to change the gloves between each procedure and another for the same patient | **30**  **(71.43%)** | **37**  **(88.09%)** | **3.27** | **0.118** |
| 14- The syringe can be re-capped using the hands, provided that caution is exercised | **42 (100%)** | **42 (100%)** | **1.00** | **1.000** |
| 15- Chlorine at a concentration of 20 cm of chlorine is used in 980 cm of water to disinfect surfaces in the case of 5% chlorine. | **37**  **(88.09%)** | **40**  **(95.24%)** | **1.29** | **0.453** |
| 16- In the case of puncture, the puncture site is not squeezed | **39**  **(92.86%)** | **41**  **(97.62%)** | **1.00** | **0.625** |
| Number of questions answered (mean) | **12.31± 1.741** | **13.17 ± 1.21** | **t = -2.657** | **0.0112** |
| Percent (%) | **76.93± 10.91** | **82.29 ± 7.90** | **T= -2.657** | **0.0112** |
